# Anatomical Variations of the Internal Carotid Artery: A Systematic Review

**DOI:** 10.7759/cureus.89435

**Published:** 2025-08-05

**Authors:** Adriano Carneiro da Costa, Lucas Rodrigues de Sousa, Vitório Augusto Alexandre Alves, Camila Ramos Martins, Laécio Leitão Batista, Antonio Alvaro Bandeira Ferraz, Nagy Habib, Moacyr Jesus Barreto de Melo Rêgo

**Affiliations:** 1 Department of Surgery, Federal University of Pernambuco, Recife, BRA; 2 Department of Anatomical Sciences, Federal University of Campina Grande, Campina Grande, BRA; 3 Department of Family and Community Medicine, Federal University of Pernambuco, Recife, BRA; 4 Department of Diagnostic Radiology, Federal University of Pernambuco, Recife, BRA; 5 Department of Surgery &amp; Cancer, Hammersmith Hospital, Imperial College, London, GBR; 6 Department of Biology, Federal University of Pernambuco, Recife, BRA

**Keywords:** anatomical variability, a systematic review, internal carotid artery (ica), neuro-surgery, skull base surgery

## Abstract

This systematic review aims to describe the anatomical variations of the internal carotid artery (ICA) and their implications for clinical practice and surgical planning. The ICA, a major vessel supplying the brain, exhibits considerable anatomical variability that can impact the safety and efficacy of procedures involving the neck region and skull base. A comprehensive search of eight databases from 2015 to 2024 yielded 379 studies, of which eight met the inclusion criteria. The reviewed studies reported common variations such as tortuosity, protrusion into the sphenoid sinus, congenital anomalies, and bony dehiscence. These findings underscore the importance of detailed preoperative imaging and thorough anatomical knowledge to reduce surgical risks and improve patient outcomes. Recognition of ICA variants remains essential for planning and performing safe and effective surgical interventions.

## Introduction and background

The internal carotid artery (ICA) is one of the main sources of blood supply to the brain and originates from the bifurcation of the common carotid artery [1,2]. After originating in the neck, the ICA passes through the carotid canal into the skull, where it follows a complex course through its extracranial and intracranial segments [3,4].

From this perspective, the anatomical variations of this artery are important in terms of the diagnosis and treatment of the associated clinical conditions, serving as a key factor in the success of surgical and clinical interventions [5]. Reportedly, such variations affect approximately 40% of the population bilaterally and are associated with congenital anomalies or arteriosclerotic disease [6]. Among such variations in the ICA path, the most frequent is tortuosity, which is related to a reduction in the distance between the carotid bifurcation and the base of the skull, thereby increasing the risk of complications in procedures such as carotid endarterectomy [5]. Furthermore, these oscillations can also manifest as sharply angled bends, which are commonly observed in older patients or those affected by atherosclerosis [5]. Even in extreme cases, tortuosity can compress adjacent structures, generating symptoms such as dysphagia, glossopharyngeal neuralgia, and foreign body sensation in the pharynx [6].

Another important variation involves protrusion and deficiency of the ICA in relation to the sphenoid sinus, which is present in up to 35% of patients. In this analysis, involvement of the parasellar and paraclival segments of the artery is associated with a potentially increased risk of iatrogenic injuries during otorhinolaryngological surgeries and procedures at the base of the skull [7].

Clinical knowledge of such circumstances is essential to minimize complications, especially in endonasal surgeries, where there is proximity between the ICA and critical structures, as well as an increased risk of injuries [7], since protrusion of the ICA in the sphenoid sinus was identified in approximately 34.4% of the cases studied [8], highlighting the need for a detailed anatomical evaluation before surgical procedures in this location.

In rare cases, there may be a congenital absence of the ICA, a condition that can lead to the development of compensatory collateral pathways [4]. This absence also causes significant changes in cerebral blood circulation, increasing the likelihood of aneurysms and other vascular complications [4,8,9]. Associated symptoms may present as ischemic events or subarachnoid hemorrhages, depending on the severity of the anatomical and hemodynamic anomalies [4]. In some cases, ICA variations with complex courses may extend into regions such as the parapharyngeal space, increasing surgical risk [6,10]. For instance, the presence of a unilateral thyrovertebral trunk, as reported in a recent cadaveric study by Wong [10], underscores the embryological basis of arterial diversity and its potential clinical implications. These findings provide further context for understanding the origin-site deviations of the ICA and reinforce the importance of preoperative vascular mapping to prevent inadvertent vascular injury.

Thus, based on the issues outlined, the objective of this review is to assess the recognition of ICA anatomical variations. This study is essential for both surgical analysis and planning, as well as for preventing possible complications and risks during invasive procedures in the cervical region and at the base of the skull [7]. Therefore, evaluation using computed tomography (CT) and magnetic resonance imaging (MRI) is important for identifying critical variants and planning safe access during invasive surgeries [8].

## Review

Methodology

Study Design

This is a systematic review conducted in accordance with the Preferred Reporting Items for Systematic Reviews and Meta-Analyses (PRISMA) guidelines [11], aiming to synthesize available evidence on anatomical variations of the ICA in humans.

Search Strategy

A comprehensive electronic search was performed in September 2024 across eight databases: PubMed, Scopus, Embase, SciELO, ScienceDirect, Cochrane Library, SpringerLink, and Latindex. Articles published in English between 2015 and 2024 were eligible for inclusion. Descriptors were selected using the DeCS (Health Sciences Descriptors) vocabulary, and the following Boolean combination was applied: “Anatomical variation” AND “Internal carotid artery” AND “Skull.” Duplicate records were removed using Rayyan software (Rayyan Systems Inc., Cambridge, MA, US) to ensure that each study was counted only once.

Eligibility Criteria

Original studies involving anatomical variations of the ICA in humans, with relevant titles and abstracts addressing variability of this artery, were included. Studies unrelated to the ICA or without clear anatomical relevance, as well as non-human studies, reviews, case reports, and conference abstracts, were excluded.

Study Selection

The data extraction and review process was carried out by pairs of independent reviewers using structured instruments and a predetermined consensus process [12,13]. All authors participated in a calibration exercise, in which they independently reviewed a sample paper and then discussed each evaluation item to align understanding and interpretation.

When discrepancies arose during independent assessments, the following consensus strategy was applied [13]: First, reviewers determined whether disagreements stemmed from factual oversights or subjective interpretation. Factual discrepancies-most commonly due to omissions or misreadings-were resolved by rechecking the original manuscript. If reviewers disagreed on the extent to which an item was met, they discussed whether the item had been adequately documented. If disagreement persisted by one point, the lower score was assigned by default. If disagreement exceeded one point, an adjudicator would be involved; however, this was not necessary during the study.

Inclusion Criteria

Studies were considered eligible if they met the following inclusion criteria. Eligible articles included original research reporting anatomical variations of the ICA in human subjects, with titles and abstracts clearly addressing ICA variability. Only peer-reviewed studies published in English between 2015 and 2024 were considered.

Exclusion Criteria

Studies were excluded if they did not specifically address the ICA or lacked anatomical relevance. Additional exclusion criteria included non-human studies, narrative or systematic reviews, meta-analyses, case reports, letters to the editor, technical notes, editorials, conference abstracts, gray literature, or any article without accessible full text.

Evaluation Criteria

The articles were critically analyzed through an interpretation guide used to evaluate their individual quality, based on the study [12] and adapted by MacDermid et al. [13]. The articles’ quality evaluation items are expressed by scores in Table 1, in which 0 = absent, 1 = incomplete, and 2 = complete. Total score (%): 0%-100%, calculated as (Sum of scores/[2 × number of applicable items]) × 100.

**Table 1:** Analysis of the quality of studies on anatomical variations of the internal carotid artery. NA: not applicable

Study	1	2	3	4	5	6	7	8	9	10	11	12	Total
Zhang et al. (2018) [4]	2	2	2	2	1	NA	2	2	2	1	2	2	90.91%
McNamara et al. (2015) [5]	2	2	2	1	0	NA	1	2	2	1	1	2	72.73%
Bernardes et al. (2021) [6]	1	2	1	1	1	NA	2	2	2	1	0	1	63.64%
Dal Secchi et al. (2018) [7]	1	2	1	1	1	NA	2	2	2	1	0	1	63.64%
Famurewa et al. (2019) [8]	2	2	2	2	1	NA	2	2	2	1	2	2	90.91%
AlQarni et al. (2022) [9]	2	2	1	1	1	NA	1	2	2	1	2	2	77.27%
Periyasamy et al. (2024) [14]	2	1	2	1	1	NA	2	2	1	1	2	2	77.27%
Georgiadi et al. (2023) [15]	2	2	2	2	1	NA	2	2	2	1	2	2	90.91%

Each study was assessed using standardized evaluation criteria (12 items): 1: a thorough literature review to define the research question; 2: specific inclusion/exclusion criteria; 3: specific hypotheses; 4: appropriate achievement of psychometric properties; 5: sample size; 6: follow-up; 7: the authors referenced specific procedures for the administration, scoring, and interpretation of procedures; 8: the measurement techniques were standardized; 9: data are presented for each hypothesis; 10: appropriate statistical point estimates; 11: appropriate statistical error estimates; 12: conclusions and clinical recommendations (Table 1).

Statistical Analysis

Interobserver agreement was assessed using the Kappa (κ) statistic, computed in BioEstat v5.3 software (BioEstat, Belém, Brazil), following the Landis and Koch scale [16]. The resulting κ-value was 0.815, indicating substantial agreement between reviewers.

Results

Study Selection

**Figure 1:**
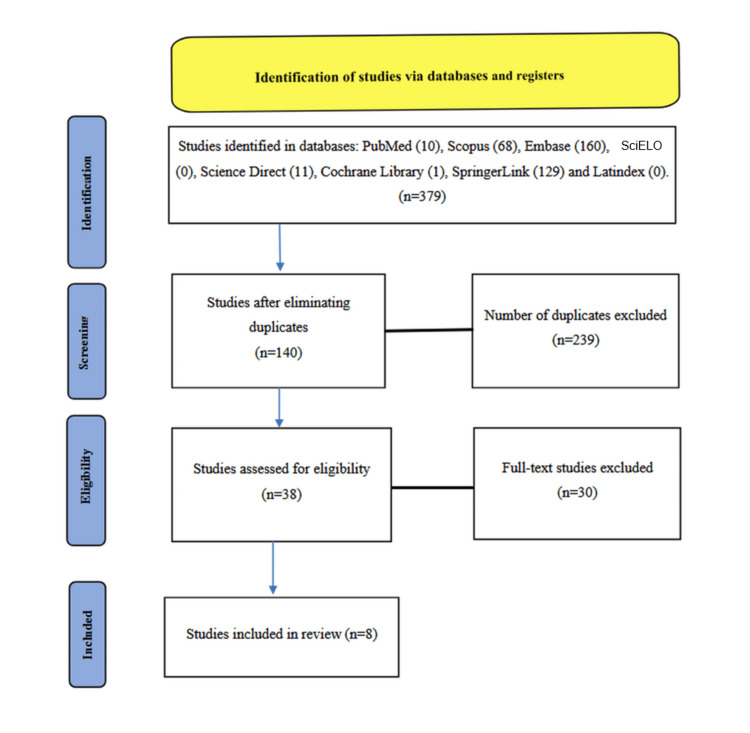
Process of searching and selecting studies for the systematic review, following PRISMA guidelines. PRISMA: Preferred Reporting Items for Systematic Reviews and Meta-Analyses

The systematic search initially identified 379 articles. After removal of duplicates (n = 239) and application of the inclusion and exclusion criteria, 38 full-text articles were assessed for eligibility. Of these, 30 were excluded due to insufficient data on anatomical variations of the ICA or lack of imaging-based anatomical descriptions. A total of eight studies were ultimately included for qualitative synthesis. The study selection process is illustrated in the PRISMA flow diagram (Figure 1).

Study Characteristics

The main characteristics of the included studies are summarized in Table 2.

**Table 2:** Main characteristics of the included studies. CT: computed tomography; MRI: magnetic resonance imaging; DSA: digital subtraction angiography; ICA: internal carotid artery

Author (year)	Country	Study design	Sample size	Imaging modality	Anatomical focus	Key findings
McNamara et al. (2015) [5]	USA	Retrospective	146	CT angiography	Tortuosity, kinking	ICA tortuosity more frequent with bifurcation < 5 cm from skull base
Dal Secchi et al. (2018) [7]	Brazil	Retrospective	92	CT	ICA protrusion (parasellar/paraclival)	Protrusion present in 26% (parasellar) and 35% (paraclival)
Famurewa et al. (2018) [8]	Nigeria	Prospective	101	MRI	Protrusion, sinus pneumatization	Correlation between sinus pneumatization and ICA protrusion (34%)
Zhang et al. (2018) [4]	China	Case-control	64	DSA	Congenital absence, collaterals	25% of congenital ICA absence associated with aneurysms
Periyasamy et al. (2024) [14]	India	Cross-sectional	178	CT	Dehiscence of ICA wall	Dehiscence more frequent in chronic rhinosinusitis
Bernardes et al. (2021) [6]	Brazil	Retrospective	87	MRI/CT	Kinking, pharyngeal compression	ICA kinking related to oropharyngeal compression risks
AlQarni et al. (2022) [9]	Saudi Arabia	Case report	1	CT angio/MRI	Congenital absence	Associated with aneurysm and ophthalmic artery anomaly
Georgiadi et al. (2023) [15]	Greece	Retrospective	30	CT	Bilateral ICA hypoplasia	Higher incidence of aneurysms

Risk of Bias and Study Quality

Study quality was assessed using a modified version of the critical appraisal tool (criteria described in Table 3). Out of the eight studies, five met ≥80% of the criteria and were considered high quality. The remaining three were considered of moderate quality due to incomplete methodology reporting or small sample size.

**Table 3:** Risk of bias and methodological quality assessment for included studies.

Study	Total score (out of 24)	Quality rating
McNamara et al. (2015) [5]	21	High
Dal Secchi et al. (2018) [7]	20	High
Famurewa et al. (2018) [8]	19	High
Zhang et al. (2018) [4]	17	Moderate
Periyasamy et al. (2024) [14]	22	High
Bernardes et al. (2021) [6]	20	High
AlQarni et al. (2022) [9]	14	Moderate
Georgiadi et al. (2023) [15]	16	Moderate

Results of Individual Studies

Tortuosity and kinking were found in five of eight studies, with McNamara et al. [5] reporting tortuosity in 42.5% of cases. Bernardes et al. [6] associated kinking with surgical complications during oropharyngeal access. ICA protrusion was noted by Dal Secchi et al. [7] and Famurewa et al. [8] in up to 35% of cases, often correlated with extensive sinus pneumatization. Dehiscence was observed in 27% of patients with chronic sinusitis by Periyasamy et al. [14]. Congenital absence and hypoplasia, though rare, were reported by Zhang et al. [4] and Georgiadi et al. [15] and are often associated with collateral circulation or aneurysms.

Synthesis of Results

Overall, the most commonly reported variations were tortuosity/kinking (62.5%), ICA protrusion into the sphenoid sinus (50%), dehiscence (12.5%), and congenital absence/hypoplasia (25%). Despite heterogeneity in study designs and classifications, the findings consistently highlight anatomical variations with potential clinical implications in the skull base and cervical surgery.

Discussion

This systematic review identified key anatomical variations of the ICA with potential clinical relevance, based on eight selected studies. The findings emphasize the importance of recognizing these variants to guide safe surgical planning, particularly in procedures involving the skull base and cervical region.

Tortuosity and Kinking

Tortuosity and kinking were the most frequently reported variations, identified in five of the included studies. McNamara et al. [5] reported a 42.5% prevalence of ICA tortuosity, with an association between high carotid bifurcation and increased intraoperative difficulty. Bernardes et al. [6] also linked kinking to complications during oropharyngeal access, reinforcing the clinical importance of identifying these patterns through preoperative imaging.

ICA Protrusion Into the Sphenoid Sinus

Two studies, by Dal Secchi et al. [7] and Famurewa et al. [8], documented ICA protrusion into the sphenoid sinus in up to 35% of cases. This variation was more frequent in patients with extensive sphenoid pneumatization and is a known risk factor for iatrogenic vascular injury during endonasal approaches such as transsphenoidal surgery [8]. Dal Secchi et al. [7] highlighted that protrusion typically involves the parasellar and paraclival segments of the ICA.

Bony Dehiscence

Dehiscence of the bony wall separating the ICA from the sphenoid sinus was described by Periyasamy et al. [14] in 27% of patients with chronic rhinosinusitis. This condition exposes the artery to direct injury during endoscopic procedures and is associated with long-standing inflammatory changes in the sinus.

Congenital Absence and Hypoplasia

Zhang et al. [4] and Georgiadi et al. [15] reported rare cases of congenital ICA absence and bilateral hypoplasia. These anomalies were associated with compensatory development of collateral circulation and a higher incidence of intracranial aneurysms. In Zhang et al. [4]’s study, 25% of patients with congenital absence presented with aneurysmal formations, while Georgiadi et al. [15] described an increased vulnerability to cerebrovascular complications. Similarly, Given et al. [17] reported that congenital ICA absence was often accompanied by collateral circulation via the circle of Willis and an aneurysm prevalence of up to 34%. Sudheer [18] described a case of ICA agenesis associated with an anterior communicating artery aneurysm, while Gupta et al. [19] documented bilateral ICA hypoplasia in a postpartum female who developed cerebrovascular complications and a posterior communicating artery aneurysm. Nicoletti et al. [20] also identified ICA hypoplasia in asymptomatic adults, highlighting the presence of extensive collateral networks and the importance of early radiologic detection to prevent neurovascular complications.

Summary of Findings

Across the eight studies, the most common anatomical variations were tortuosity/kinking (reported in 62.5% of studies), ICA protrusion into the sphenoid sinus (50%), congenital absence or hypoplasia (25%), and bony dehiscence (12.5%). These variations pose significant surgical risks and warrant thorough preoperative evaluation.

Limitations

This review is limited by methodological heterogeneity among the included studies, particularly in imaging techniques and classification criteria. Additionally, the small number of studies and the absence of meta-analysis limit the generalizability and quantitative strength of the conclusions.

## Conclusions

Anatomical variations of the ICA are frequent and clinically relevant, particularly in skull base and neck surgeries. Based on this review, we recommend routine preoperative imaging (CT or MRI) to identify ICA variants, especially in high-risk patients. Surgical planning should incorporate these findings to avoid complications, particularly in cases of tortuosity, kinking, or protrusion into the sphenoid sinus. Awareness of rare anomalies like congenital absence or hypoplasia is also essential, as they may alter cerebral circulation and increase aneurysm risk. Incorporating these measures into clinical practice can enhance surgical safety and improve patient outcomes.
